# Prevalence and Risk Factors for Diabetic Lower Limb Amputation: A Clinic-Based Case Control Study

**DOI:** 10.1155/2016/5941957

**Published:** 2016-06-29

**Authors:** Beverly T. Rodrigues, Venkat N. Vangaveti, Usman H. Malabu

**Affiliations:** Department of Diabetes and Endocrinology, The Townsville Hospital and College of Medicine and Dentistry, James Cook University, 100 Angus Smith Drive, Douglas, QLD 4814, Australia

## Abstract

*Objective.* The aim of the study was to evaluate the prevalence of and risk factors for lower limb amputation in a specialist foot clinic-based setting.* Methods.* A retrospective quantitative study was conducted, using clinical and biochemical profiles of diabetic foot patients attending the High Risk Foot Clinic at The Townsville Hospital, Australia, between January 1, 2011, and December 31, 2013.* Results.* The total study sample included 129 subjects, comprising 81 males and 48 females with M : F ratio of 1.7 : 1. Twenty-three subjects were Indigenous Australians, representing 17.8% of the study population. The average age of the cohort was 63.4 years ± 14.1 years [CI 90.98–65.89]. Lower limb amputation was identified as a common and significant outcome (*n* = 44), occurring in 34.1%, more commonly amongst the Indigenous Australians (56.5% versus 29.2%; *p* = 0.94, OR 0.94). Risk factors most closely associated with amputation included diabetic retinopathy (*p* = 0.00, OR 4.4), coronary artery bypass graft (CABG) surgery (*p* = 0.01, OR 4.1), Charcot's arthropathy (*p* = 0.01, OR 2.9), and Indigenous ethnicity (*p* = 0.01, OR 3.4). Although average serum creatinine, corrected calcium, and glycosylated haemoglobin A1c (Hba1c) levels were higher amongst amputees they were statistically insignificant.* Conclusions.* Lower limb amputation is a common outcome and linked to ethnicity and neurovascular diabetic complications amongst subjects with diabetic foot ulcer. Further research is needed to identify why risk of lower limb amputation seems to differ according to ethnicity.

## 1. Introduction

Diabetes and the diabetic foot ulcer (DFU) have made their mark in society, with the prevalence of diabetes being four times higher than all cancers combined [1]. Increased life expectancies have contributed significantly to this exponential rise, with diabetes now contributing to 9% of global mortality, equating to 4 million deaths per year [2, 3]. DFU occurs as a diabetic complication and involves a multifactorial pathogenesis including peripheral neuropathy as the primary causal factor, together with variable contribution from peripheral vascular disease (PVD), repetitive trauma, and superimposing foot infection [4, 5]. Infected DFU is a major cause of prolonged hospital admission and contributes over 90% of nontraumatic lower limb amputations (LLAs) [6, 7], which is more than a million amputations/year [8–10]. Whilst the 1990 St. Vincent Declaration to half LLAs in 5 years has failed [11], we have instead seen a 50% 5-year mortality rate amongst diabetic amputees [12–14].

The progressive rise of diabetes is likely to pose a significant burden on future society leading to an associated increase in diabetic amputations [15, 16]. Despite previous alert to the importance of early detection and management, prevention practices remain poor, with inconsistent patient follow-up and management compliance [17, 18]. As a result, subjects with DFU maintain poorer quality of life, with higher baseline depression rate, and 5-year mortality rates of up to 74% [19]. Existing studies have identified Indigenous ethnicity and presence of microvascular complications as contributing factors to poor DFU outcomes; however there is currently limited Australian evidence supporting this [20]. Furthermore there is no current data on the burden of either amputation or macrovascular outcomes amongst subjects with DFU in clinic setting in Australia which is home to amongst others Indigenous population at risk of diabetes. This study endeavoured to bridge these current knowledge gaps, with particular focus on determining prevalence of and identifying risk factors of limb amputations amongst patients with DFU attending a typical Australian regional diabetes foot clinic.

## 2. Methods

### 2.1. Eligibility Criteria

All patients attending the High Risk Foot Clinic (HRFC) at Northeastern Australia's Townsville Hospital between January 1, 2011, and December 31, 2013, were included in the study. Patients 18 years of age and over with a confirmed diagnosis of either type 1 or type 2 diabetes and coexisting DFU were included. Subjects under the age of 18 and those with nondiabetic foot ulcers (e.g., trauma-related, vasculitic, and neoplastic ulcers) were excluded from the study. Subjects who attended the clinic for other management purposes (e.g., podiatry reviews, nail pathology, and education about prevention) were also excluded.

### 2.2. Data Extraction

There were two main processes involved in data extraction, including gathering of clinical and biochemical data from patient profile. A retrospective chart audit was initially performed, focusing on the correspondence and outpatient attendance sections. The HRFC pro forma sheet was then used to collect information regarding the onset, duration, outcome, and type of ulcer defined as ischaemic or nonischaemic based on University of Texas Wound Classification System [21]. Furthermore, clinical information on the diagnosis and management of diabetes, coexisting macrovascular and microvascular complications, and medication lists of the subjects was recorded. We also obtained biochemical data using the hospital's pathology system, AUSLAB. Main parameters gathered included results of full blood count, urea and electrolyte studies, lipid profiles, Hba1c levels, and serum calcium, phosphate, and C-reactive protein levels. Average figures over the three-year period were calculated and included.

### 2.3. Data Analysis

SPSS software was utilised to perform data analysis. Basic descriptive and frequency analyses of the study sample were implemented to obtain demographic characteristics, period prevalence of clinical outcomes and diabetic complications, and mean age of the study population. In addition, a combination of nonparametric and chi-squared analyses was performed to identify differences in scaled data and rank risk factors associated with amputation, respectively. *p* value of less than 0.05 was considered statistically significant and together with odds ratios and confidence intervals was included in our results. All significant values were entered into binary logistic regression analysis and correction for multiple regression logistic testing was then conducted and factored in as part of our final results.

## 3. Results

### 3.1. Study Characteristics

A total of 129 subjects were included in the analysis of this study, with 62.8% being male (*n* = 81) and 37.2% female (*n* = 48) (refer to Table 1). The mean age of the study cohort was 63.4 ± 14.1 years [CI 60.98–65.89]. The Indigenous cohort comprised 17.8% (*n* = 23). Patients were categorised according to comparison groups of ischaemic (*n* = 57) and nonischaemic (*n* = 72) ulcers. The period prevalence of amputation within the study sample was 34.1% (*n* = 44), with 35.1% belonging to ischaemic (*n* = 20) and 33.3% nonischaemic (*n* = 24) cohorts. Amputation occurred more commonly at a rate of 56.5% amongst the Indigenous subjects, in comparison with 29.2% in the non-Indigenous group, with a significant difference amongst the ischaemic and nonischaemic ulcer cohorts (69.2% versus 30.8%). The mean age of amputation of 62.6 ± 12.5 years [CI 55.09–70.14] amongst the Indigenous cohort was similar to their non-Indigenous counterparts, mean age of 62.0 ± 11.5 years [CI 57.81–66.25]. Amputation rates were much higher amongst patients with PVD (39.4% versus 20%). Males got amputated more frequently (59.1%) compared to females (40.9%), although the mean age of male and female amputees differed minimally at 61.0 years and 63.9 years, respectively. Prevalence of ischaemic heart disease (IHD), acute myocardial infarcts (AMI), CABG surgery, strokes, and dialysis was significantly higher amongst ischaemic ulcer patients, occurring at overall rates of 54.4%, 26.3%, 15.8%, 19.3%, and 7.0%, respectively.

### 3.2. Biochemical Parameters amongst the Study Cohort

Using nonparametric analysis, corrected calcium levels were calculated to be higher amongst the amputee cohort (2.37 mmol/L versus 2.26 mmol/L (*p* = 0.003)) (refer to Table 2) though clinically nonsignificant with normal range 2.15–2.60 mmol/L. Similarly, although amputees were found to have higher serum creatinine (156.21 *μ*mol/L versus 120.91 *μ*mol/L) and Hba1c (9.0% versus 8.5%) levels, these results were statistically insignificant (*p* = 0.19 and *p* = 0.44). Indigenous subjects had lower average white cell counts (*p* = 0.005) and higher albumin levels (*p* = 0.058). Males tended to have poorer kidney function than females, with average creatinine levels of 155.03 *μ*mol/L versus 99.25 *μ*mol/L (*p* = 0.003) and eGFR levels of 57.94 mL/min versus 63.90 mL/min (*p* = 0.22).

### 3.3. Clinical Risk Factors Associated with Amputation

Chi-squared analysis identified diabetic retinopathy (*p* = 0.00, OR 4.4 [2.15–12.75]), Indigenous background (*p* = 0.01, OR 3.1 [1.17–9.16]), Charcot's arthropathy (*p* = 0.01, OR 2.9 [1.38–9.29]), longer diabetes duration, defined as 15 years or longer (*p* = 0.03, 2.2 [1.00–4.86]), dyslipidaemia (*p* = 0.04, 3.4 [0.94–12.38]), neuropathy (*p* = 0.03, 3.3 [1.05–10.26]), PVD (*p* = 0.04, 2.6 [1.03–6.55]), and CABG surgery (*p* = 0.01, OR 4.1 [1.81–30.76]) to be significantly associated with increased risk of amputation, amongst others (refer to Table 3). Binary logistic regression analyses identified retinopathy, CABG surgery, Charcot's arthropathy, and Indigenous background as the most significant risk factors for amputation (refer to Table 4). Clinical parameters that fell short of statistical significance include smoking history (*p* = 0.49), dialysis (*p* = 0.62), diabetes type (*p* = 0.46), male sex (*p* = 0.53), multiple-ulcer history (*p* = 0.15), previous history of DFUs (*p* = 0.06), and ulcer type (*p* = 0.84) and grade (*p* = 0.93).

## 4. Discussion

### 4.1. Prevalence of Lower Limb Amputations

In this clinic-based case control study we have demonstrated prevalence of amputation in our study population to be 34.1%, a figure that is considerably higher in comparison with others' findings of 15.4% to 21.4% [22, 23]. On the other hand our report conforms to Miner and Kirsner and Amogne et al. report of higher rate of LLA in their respective diabetic foot clinic populations [13, 24]. The high prevalence might be related to inclusion of the population at the highest risk of the disease and its complications [25]. In this study, Indigenous Australians were found to be at greater risk of diabetic LLA, which is in keeping with others' observation [20, 26]. Furthermore, whilst there was a marginal difference in amputation between ischaemic and nonischaemic cohorts in the overall group, amputations related to ischaemic ulcers were more than double amongst the Indigenous subgroup. Essentially, the prevalence of amputation amongst our subjects stood at comparatively higher numbers and occurred predominantly amongst Indigenous subjects with ischaemic ulcers.

### 4.2. Risk Factors for Lower Limb Amputations

We identified numerous clinical factors that correlate with higher amputation risk, most in keeping with previous literature, in addition to new, undocumented parameters. The most significant contributing factors were diabetic retinopathy, CABG surgery, Charcot's foot, and Indigenous ethnicity. Whilst Ndip et al. provide data linking diabetic retinopathy to increased risk of DFU development [27], ours is the first study to identify retinopathy not only as a contributing factor, but also as the most significant factor leading to amputation amongst DF patients, accentuating the importance of early detection and management of diabetic complications. McEwen et al. found no association with retinopathy but reported a 2-3-fold amputation risk amongst patients with diabetic neuropathy [28], results that are consistent with ours, which isolate neuropathy and dyslipidaemia as significant risk factors for amputation. We identified PVD with increased risk of adverse outcome, also supported by current literature, which labels it as a causal factor for amputation and mortality amongst diabetics [29–32]. We have additionally extended existing knowledge by highlighting CABG surgery as a novel risk factor for amputation, which supports the role of both microvascular and macrovascular disease as significant risk factors for LLA in subjects with diabetic foot ulcer.

Interestingly, there has been conflicting data on the role of ethnicity in the outcome of DFUs. Whilst multiple studies found unremarkable figures in amputation rates amongst a multiracial cohort [27, 28], Lavery et al. found a significant difference in both prevalence and ulcer severity contributing to amputation amongst African American cohorts in other parts of the world [33]. Correspondingly, we have found Indigenous ethnicity to be amongst the strongest contributing factors in our cohort, who were almost twice as likely to undergo an amputation. The higher prevalence of amputations in the group of Indigenous Australians could be attributed to a genetic predisposition or to a socioeconomic status that drives the patients to present late for clinical care. This result is supported by previous Australian data stating that Indigenous Australians are known to develop diabetes and its associated metabolic complications at a younger age [24, 34]. As the first study to be conducted in Northeastern Australia which hosts some of the largest Indigenous peoples nationwide, our results hold considerable significance for the local population, given that longer diabetes duration was flagged as a contributor for adverse outcome and highlights the need for earlier detection and management amongst Indigenous Australians [32, 35, 36].

Our results identified other risk factors of LLA in subjects with DFU including Charcot's arthropathy, a history of osteomyelitis, and severity of foot infection or cellulitis requiring antibiotic treatment. Our result was in keeping with the previously reported 30% rate of amputation in subjects with Charcot's arthropathy, placing DFU patients at a 12 times higher lifetime risk of amputation [37, 38]. Similarly, in keeping with our findings, Wukich et al. have linked history of cellulitis and moderate-to-severe foot infection to amputation [39]. There is notably no current evidence suggesting use of antibiotics to prevent infections in subjects with DFU at risk of LLA in spite of our findings of antibiotics use preceding amputation. The tropical climate of Northeastern Australia is a likely contributor to this association, resulting in increased rates of bacterial skin and soft tissue infections requiring ongoing antimicrobial treatment. Moreover, a new association was also found with depression, suggesting that psychological health can be an indicator of healing and recovery in physical illness. Given that the DFU is known to have a biopsychosocial impact on its patients, this information accents the importance of a multidisciplinary team approach in treating the individual as a whole and calls for additional research in the area.

In contrast to others' observation, we found no association between renal disease and amputation risk, specifically with CKD and dialysis, all of which have previously been linked with amputation [35, 40]. Amongst our dialysis cohort, all seven subjects were male, with one having Indigenous background. These characteristics were similar to Lavery et al.'s study, whose dialysis cohort mainly focused on a non-Hispanic Caucasian population, yet whilst they identified more limb amputations amongst their renal disease subgroup, we found no significant association between the two [41]. This could be explained by the nature of our study cohort, which focused on DFU patients with concurrent renal disease rather than a specific CKD or dialysis population, thereby missing a number of diabetic LLA patients that either did not meet the criteria or failed to attend the HRFC. Intensive glycaemic control has been established as an important prognostic healing factor and found to reduce diabetes-related mortality [42, 43]. However when discussed in linkage with lower extremity complications, some studies report a 20% higher risk of amputation amongst subjects with Hba1c levels above 7.5% similar to our findings but in contrast to Winkley et al. who reported Hba1c levels below 7.5% as having higher mortality and increased risk of amputation [31, 32, 44]. In light of this conflicting report further research is required to characterise the findings.

The period prevalence of IHD and AMI amongst the study cohort was similar to others' report indicating high prevalence of cardiovascular disease and its complications amongst the DF cohort [45]. CKD and dialysis were present in 40.3% and 5.4%, which was once again lower compared to international data and again likely due to the fact that ours was not a renal-specific cohort. We did, however, establish higher rate of PVD (72.9%) compared to Setacci et al., who reported a period prevalence of up to 30% amongst the diabetic cohort [46], which reinforces the role of PVD in contributing to amputation. Furthermore, 14.7% of the population had a background history of strokes, with no previous studies available with which to make comparisons. Our results have appropriately highlighted that the high rates of adverse multisystemic, vascular outcomes amongst patients with the DF necessitate a multidisciplinary approach in treatment delivery.

### 4.3. Strengths and Limitations

Major study strength included the data extraction process, which utilised a wide range of demographic, clinical, and biochemical data to formulate an extensive analysis to support the study aims. This study implemented a highly focused study cohort with a good representation of Indigenous subjects to evaluate and compare our results with international data. It is important to note that there is a limitation to retrospective studies in general. Observations derived from such studies may contain some missing information and thus may serve as a stimulus to further prospective work to clarify findings. The present work must be interpreted in the knowledge of the defects inherent in such studies. Nevertheless, our result is in agreement with other reports [20, 22, 47].

## 5. Conclusions

We have documented high prevalence of lower limb amputation in our study population. In keeping with our hypothesis, the ischaemic ulcer cohort demonstrated higher rates of adverse clinical outcomes, including IHD, strokes, and renal disease. Numerous known and novel demographic and clinical risk factors were coupled with amputation, the most significant of them being Indigenous ethnicity, diabetic retinopathy, Charcot's arthropathy, and CABG surgery. Whilst there were no significant differences in amputation prevalence between the ischaemic and nonischaemic groups in the overall cohort, Indigenous subjects with ischaemic ulcers were amputated much more frequently. Extended research in the local area is encouraged to study factors leading to selectively higher amputation rate in the Indigenous population. We have made a huge development in identifying predisposing characteristics amongst DF patients, but our knowledge is not yet comprehensive to enable us to prevent limb amputation in the high risk diabetic population.

## Figures and Tables

**Table 1:** Basic study characteristics and prevalence of adverse outcomes amongst the diabetic foot ulcer cohort.

Characteristic	Results			
Age	63.43 years ± 14.07 years [CI 60.98–65.89]			
Sex	Males (*n* = 81; 62.8%); females (*n* = 48; 37.2%)			
Ethnicity	Indigenous (*n* = 23; 17.8%); non-Indigenous (*n* = 106; 82.2%)			
Type of diabetes	Type 1 (*n* = 22; 17.1%); type 2 (*n* = 107; 82.9%)			
Type of ulcer	Ischaemic (*n* = 57; 44.2%); nonischaemic (*n* = 72; 55.8%)			

Clinical outcome	Total study sample	Ischaemic ulcer cohort	Nonischaemic ulcer cohort	*p* value, OR [CI]

Amputation	(*n* = 44)—34.1%	(*n* = 20)—35.1%	(*n* = 24)—33.3%	*p* = 0.84, 1.1 [0.52–2.25]
Minor amputation	(*n* = 35)—27.13%	(*n* = 15)—42.9%	(*n* = 20)—57.1%	*p* = 0.50, 0.9 [0.14–2.62]
Major amputation	(*n* = 9)—6.98%	(*n* = 5)—55.5%	(*n* = 4)—44.4%	*p* = 0.50, 1.5 [0.46–4.84]
Indigenous amputations	(*n* = 13)—56.5%	(*n* = 9)—69.2%	(*n* = 4)—30.8%	*p* = 0.94, 0.9 [0.17–5.07]
Ischaemic heart disease	(*n* = 61)—47.3%	(*n* = 31)—54.4%	(*n* = 30)—41.7%	*p* = 0.15, 1.7 [0.83–3.36]
Acute myocardial infarction	(*n* = 27)—20.9%	(*n* = 15)—26.3%	(*n* = 12)—16.7%	*p* = 0.18, 1.8 [0.76–4.20]
Cerebrovascular accidents	(*n* = 19)—14.7%	(*n* = 11)—19.3%	(*n* = 8)—11.1%	*p* = 0.19, 1.9 [0.71–5.13]
Chronic kidney disease	(*n* = 52)—40.3%	(*n* = 27)—47.4%	(*n* = 25)—34.7%	*p* = 0.20, 1.6 [0.78–3.23]
Dialysis	(*n* = 7)—5.4%	(*n* = 4)—7.0%	(*n* = 3)—4.2%	*p* = 0.48, 1.7 [0.37–8.09]
Peripheral vascular disease	(*n* = 94)—72.9%	(*n* = 44)—77.2%	(*n* = 50)—69.4%	*p* = 0.33, 1.5 [0.67–3.30]

**Table 2:** Summary of biochemical parameters amongst the study cohort.

Biochemical variable	Cohort		*p* value
	*Ischaemic ulcers*	*Nonischaemic ulcers*	
Average Hba1c level	8.5%	9.0%	*p* = 0.06
Average CRP level	38.6	72.7	*p* = 0.14

	*Amputees*	*Nonamputees*	
Average Hba1c level	9.0%	8.5%	*p* = 0.44
Average correct Ca^2+^ level	2.4 mmol/L	2.3 mmol/L	^*∗*^ *p* = 0.00
Average serum creatinine level	156.2 *μ*mol/L	121 *μ*mol/L	*p* = 0.19

	*Males*	*Females*	
Average serum creatinine level	155 *μ*mol/L	99.3 *μ*mol/L	^*∗*^ *p* = 0.00
Average eGFR level	57.4 mL/min	63.9 mL/min	*p* = 0.22

	*Indigenous*	*Non-Indigenous*	
Average white cell count	7.4	8.9	^*∗*^ *p* = 0.01
Average albumin level	37	34.2	*p* = 0.06

^*∗*^Statistically significant.

**Table 3:** Summary of risk factors for lower limb amputation amongst the cohort using chi-squared analysis.

Risk factor	*p* value, OR [CI]
Acute myocardial infarction	*p* = 0.20, 1.8 [0.74–4.16]
Antihypertensive medications	*p* = 0.28, 1.8 [0.62–5.32]
Cellulitis	^*∗*^ *p* = 0.00, 3.3 [1.54–7.21]
Cerebrovascular accidents	*p* = 0.07, 2.5 [0.93–6.67]
Charcot's arthropathy	^*∗*^ *p* = 0.01, 2.9 [1.29–6.70]
Chronic kidney disease	*p* = 0.27, 1.5 [0.72–3.16]
Coronary artery bypass graft surgery	^*∗*^ *p* = 0.01, 4.1 [1.29–13.17]
Depression	*p* = 0.05, 2.2 [0.98–5.10]
Dialysis	*p* = 0.62, 1.5 [0.32–6.94]
Dyslipidaemia	^*∗*^ *p* = 0.04, 3.4 [0.94–12.38]
eGFR < 45 mL/min	*p* = 0.08, 2.1 [0.91–4.73]
Foot antibiotics	^*∗*^ *p* = 0.04, 2.3 [1.03–4.98]
Gastrooesophageal reflux disease (GORD)	*p* = 0.34, 1.5 [0.67–3.15]
Haemoglobin < 8 g/dL	*p* = 0.46, 1.7 [0.42–6.64]
Hba1c > 7.5%	*p* = 0.92, 1.1 [0.40–2.73]
Hypertension	*p* = 0.41, 1.6 [0.50–5.43]
Hypoalbuminaemia	*p* = 0.73, 0.9 [0.37–1.99]
Indigenous ethnicity	^*∗*^ *p* = 0.01, 3.1 [1.25–7.92]
Infection severity (mod-severe)	^*∗*^ *p* = 0.02, 0.4 [0.18–0.89]
Insulin treatment	*p* = 0.12, 1.9 [0.85–4.28]
Ischaemic heart disease	*p* = 0.24, 1.6 [0.75–3.24]
Ischaemic ulcer type	*p* = 0.84, 1.1 [0.52–2.25]
Longer duration of diabetes	^*∗*^ *p* = 0.03, 2.2 [1.00–4.86]
Male sex	*p* = 0.53, 0.8 [0.37–1.66]
Multiple ulcers	*p* = 0.15, 0.6 [0.25–1.25]
Nephropathy	*p* = 0.15, 1.7 [0.82–3.56]
Neuropathy	^*∗*^ *p* = 0.03, 3.3 [1.05–10.26]
Obesity	*p* = 0.09, 0.5 [0.23–1.13]
Osteomyelitis	^*∗*^ *p* = 0.00, 3.9 [1.54–10.07]
Peripheral vascular disease	^*∗*^ *p* = 0.04, 2.6 [1.03–6.55]
Previous history of ulcers	*p* = 0.06, 2.1 [0.98–4.41]
Retinopathy	^*∗*^ *p* = 0.00, 4.4 [1.99–9.59]
Smoking history	*p* = 0.49, 0.8 [0.37–1.60]
Statin therapy	*p* = 0.06, 2.7 [0.95–7.78]
Type of diabetes	*p* = 0.46, 1.4 [0.56–3.65]
Wound classification	*p* = 0.93, 1.1 [0.30–3.78]

*∗* denotes being statistically significant.

**Table 4:** Risk factors associated with lower limb amputation in the study population [logistic regression analysis].

Risk factor	*p* value, OR [CI]
Coronary artery bypass graft surgery	*p* = 0.01, 7.5 [1.81–30.76]
Indigenous ethnicity	*p* = 0.02, 3.3 [1.17–9.16]
Charcot's arthropathy	*p* = 0.01, 3.6 [1.38–9.29]
Retinopathy	*p* = 0.00, 5.2 [2.15–12.75]
